# Disability-Adjusted Life Years (DALY) for Cancer in Japan in 2000

**DOI:** 10.2188/jea.JE20110017

**Published:** 2011-07-05

**Authors:** Truong-Minh Pham, Tatsuhiko Kubo, Yoshihisa Fujino, Kotaro Ozasa, Shinya Matsuda, Takesumi Yoshimura

**Affiliations:** 1 Department of Epidemiology, Radiation Effects Research Foundation, Hiroshima, Japan; 2 Department of Preventive Medicine and Community Health, School of Medicine, University of Occupational and Environmental Health, Kitakyushu, Japan; 3 Department of Food and Health Sciences, International College of Arts and Sciences, Fukuoka Women’s University, Fukuoka, Japan

**Keywords:** cancer, DALY, disability-adjusted life years, premature mortality, years lived with disability

## Abstract

**Background:**

We used disability-adjusted life years (DALY) to estimate the cancer burden in Japan for the year 2000.

**Methods:**

We estimated years of life lost (YLL) by using mortality data and years lived with disability (YLD) by using incidence data. The DALY for cancer was calculated as the sum of YLL and YLD.

**Results:**

For all cancers combined, 2 733 884 years of DALY were estimated in men and 2 091 874 years were estimated in women. Among men, stomach and lung cancers accounted for the largest proportions of DALY, followed by liver cancer and colorectal cancer. Among women, the greatest contributors to DALY were stomach, colorectal, breast, and lung cancers.

**Conclusions:**

The national cancer burden in Japan was expressed in terms of DALY, which might be useful in assessing future changes with respect to mortality and morbidity in Japan.

## INTRODUCTION

In Japan, cancer overtook stroke in the early 1980s as the leading cause of death.^1^ Incidence and mortality rates are routinely used to quantify the cancer burden, but these measures are often reported and analyzed separately. Disability-adjusted life years (DALY) was developed in the 1990s for the Global Burden of Disease (GBD) study. Details of the GBD study have been described in textbooks^2^^,^^3^ and in a series of published articles.^4^^–^^7^ DALY describes the loss of healthy years of life, ie, the difference between actual and perfect health. It uses time units to estimate disease burden by combining years of life lost (YLL) due to premature mortality with years lived with disability (YLD) in incident cases.^2^^,^^3^

In the present study, we estimated the national cancer burden in Japan for the year 2000 by using a summary health measure to account for the burden of both mortality and incidence.

## METHODS

We calculated the DALY for cancer using procedures derived from those described in the GBD study,^2^^,^^3^ which summed the YLL and YLD components. The basic formula is expressed as follows:

DALY=YLL+YLD

We computed YLL by multiplying the number of cancer deaths by the number of years of expected remaining life at the respective age of death according to the Japanese life tables for the year 2000, in which life expectancy at birth was 85 years for women and 78 years for men.^8^ Mortality data on cancer for the year 2000 were obtained from the Vital Statistics of Japan.^9^

Next, we computed YLD by multiplying the numbers of cancer incidence by both the average duration (in years) of each cancer and a disability weight that reflected the severity of each cancer on a scale from 0 (perfect health) to 1 (death). Nationwide cancer incidence data in 2000 were obtained from estimates of a previous study.^10^ We used average durations and disability weights for countries categorized as Established Market Economies in the GBD study,^2^^,^^3^ as this information was not available for Japan.

## RESULTS

Table 1 shows the age-standardized rates (ASRs) per 100 000 according to the World Standard Population of incidence and mortality for cancer in Japan for the year 2000. Among men, there were 310 118 incident cases and 179 140 deaths, which respectively corresponded to an age-standardized incidence rate of 263.9 cases and an age-standardized mortality rate of 147.2 deaths per 100 000. Among women, there were 228 215 cases and 116 344 deaths, which corresponded to age-standardized incidence and mortality rates of 172.7 and 72.9 per 100 000, respectively.

**Table 1.** Age-standardized incidence and mortality rates per 100 000 for cancer in Japan in 2000

Cancer site	ICD-10	Men				Women			
	
		Incidence		Mortality		Incidence		Mortality	
			
		Number	ASR^a^	Number	ASR^a^	Number	ASR^a^	Number	ASR^a^
All sites	C00–96, D05–06	310 118	263.9	179 140	147.2	228 215	172.7	116 344	72.9
Mouth and pharynx	C00–C14	6650	6.1	3610	3.2	2825	2.1	1456	0.9
Esophagus	C15	13 033	11.1	8706	7.4	2418	1.5	1550	0.9
Stomach	C16	68 992	58.7	32 798	26.7	33 793	22.4	17 852	10.8
Colorectum	C18–C21	54 431	46.9	20 002	16.6	37 706	25.3	16 201	9.5
Liver	C22	27 411	23.6	23 602	20.2	12 642	7.8	10 379	6.0
Gallbladder	C23–24	8063	6.3	6913	5.4	9175	4.8	8240	4.1
Pancreas	C25	10 967	9.1	10 380	8.6	9078	5.1	8714	4.9
Larynx	C32	3250	2.8	958	0.8	209	0.1	88	0.1
Lung	C33–34	48 184	38.3	39 053	30.7	19 706	12.3	14 671	8.4
Skin	C43–44	3461	2.9	502	0.4	3398	2.0	484	0.3
Breast	C50, D05	—	—	—	—	37 389	36.7	9171	8.1
Cervix uteri	C53	—	—	—	—	7868	8.1	2393	2.0
Corpus uterus	C54–C55	—	—	—	—	6737	12.6	2809	1.9
Ovary	C56	—	—	—	—	7490	7.0	3993	3.2
Prostate	C61	19 825	14.9	7514	5.4	—	—	—	—
Kidney and urinary organs	C64–C68	17 338	14.6	6266	4.9	7199	4.4	3142	1.6
Central nervous system	C70–C72	2204	2.5	857	1.0	2188	2.2	699	0.7
Thyroid	C73	1642	1.6	411	0.3	6246	6.1	887	0.5
Hodgkin’s disease and lymphoma	C81–C85, C96	7374	6.9	4616	4.0	5933	4.6	3366	2.1
Multiple myeloma	C88–90	2140	1.7	1736	1.4	1980	1.2	1625	0.9
Leukemia	C91–C95	4578	5.1	3970	3.9	3310	3.3	2796	2.3
Other		10 575	10.8	7246	6.3	10 925	3.1	5828	3.7

^a^Age-standardized rates (ASRs) were estimated using the World Standard Population.

Table 2 shows DALY for cancer in Japan for the year 2000: 2 733 884 years in men and 2 091 874 years in women. For most specific cancer sites, YLL contributed to more than 90% of total DALY. In men, lung and stomach cancers accounted for the largest proportions of the burden, at 19.2% and 18.6% of total DALY, respectively, followed by liver cancer at 14.1% and colorectal cancer at 12.4%. Prostate cancer accounted for only 3.0%. DALY per 1000 men was 44.4 years for all cancers combined, among which the highest values were 8.5 years per 1000 men for lung cancer and 8.3 years for stomach cancer, followed by liver cancer and colorectal cancer (6.2 and 5.5 years, respectively). In women, the burden was greatest for stomach cancer, which accounted for 14.7% of total DALY, followed by colorectal cancer at 13.8%, breast cancer at 11.9%, and lung cancer at 11.2%. DALY per 1000 women for all cancers combined was 32.7 years. The highest DALY values per 1000 women were 4.8 years for stomach cancer and 4.5 years for colorectal cancer.

**Table 2.** Disability-adjusted life years (DALY) for cancer in Japan in 2000

Cancer site	Men				Women			
	
	DALY	% of total DALY	DALY per 1000 men	% of YLL in DALY	DALY	% of total DALY	DALY per 1000 women	% of YLL^a^ in DALY
All sites	2 733 884	100.0	44.4	93.8	2 091 874	100.0	32.7	95.4
Mouth and pharynx	64 993	2.4	1.1	93.7	25 883	1.2	0.4	93.5
Esophagus	141 636	5.2	2.3	96.6	25 455	1.2	0.4	95.4
Stomach	509 553	18.6	8.3	90.3	307 972	14.7	4.8	94.1
Colorectum	339 949	12.4	5.5	85.4	288 220	13.8	4.5	89.3
Liver	384 265	14.1	6.2	96.9	162 507	7.8	2.5	98.5
Gallbladder	87 439	3.2	1.4	98.6	109 379	5.2	1.7	99.4
Pancreas	158 192	5.8	2.6	96.9	133 994	6.4	2.1	98.1
Larynx	12 980	0.5	0.2	95.8	1374	0.1	0.1	96.7
Lung	524 981	19.2	8.5	97.5	234 379	11.2	3.7	97.1
Skin	8317	0.3	0.1	92.0	7922	0.4	0.1	91.8
Breast	—	—	—	—	248 765	11.9	3.9	95.5
Cervix uteri	—	—	—	—	60 296	2.9	0.9	96.5
Corpus uterus	—	—	—	—	54 796	2.6	0.9	96.3
Ovary	—	—	—	—	94 439	4.5	1.5	97.8
Prostate	81 657	3.0	1.3	87.9	—	—	—	—
Kidney and urinary organs	85 828	3.1	1.4	92.6	44 556	2.1	0.7	94.7
Central nervous system	22 129	0.8	0.4	96.1	20 577	1.0	0.3	96.0
Thyroid	6266	0.2	0.1	89.8	14 998	0.7	0.2	84.0
Hodgkin’s disease and lymphoma	75 161	2.8	1.2	96.7	60 831	2.9	0.9	96.9
Multiple myeloma	24 017	0.9	0.4	96.8	25 369	1.2	0.4	97.4
Leukemia	85 548	3.1	1.4	94.6	68 487	3.3	1.1	95.1
Other	120 973	4.4	2.0	97.4	101 675	4.9	1.6	97.5

^a^YLL denotes years of life lost.

## DISCUSSION

We estimated the cancer burden in Japan for the year 2000 by using DALY to account for the burden of both cancer incidence and mortality. The results reflect the contributions of both measures to total cancer burden.

Because cancer is a potentially fatal condition, YLL was the predominant contributor to DALY estimates in the present study, although for most cancer sites the number of incident cases was approximately twice that of deaths. This is consistent with other studies.^11^^,^^12^ A study in France reported that YLL contributed to 98% of DALY for lung cancer in men and 86% of DALY for breast cancer in women,^11^ while a study in Australia showed a greater than 80% contribution of YLL to total DALY for all cancer.^12^ A study estimating the cancer burden in Spain in 2000 reported 84% of DALY for cancer (both sexes combined).^13^ In contrast, for less fatal conditions the impact of YLD might be greater than that of YLL. The DALY for mental disorders and musculoskeletal disease in studies in Australia, for instance, was mainly attributable to YLD.^12^^,^^14^ In addition, differences among studies in the YLL/YLD ratio for DALY might be due to variations in the age structure of the respective populations, the incidence/mortality ratio, or average age at onset or death.

In the GDB study, social preferences regarding age weighting and the discount rate for future years were considered in the computation of DALY.^2^^,^^3^ The discount rate emphasizes the social value of a healthy year now rather than in the future, while age weighting reflects the fact that a year of life in young adulthood is more valued than a year of life in old age or infancy.^2^ However, in order to provide actual estimates, we used neither discounting nor age weighting in the present study. Moreover, the use of these adjustments is controversial.^2^^,^^12^^,^^15^^,^^16^ Indeed, a recent Dutch study used neither,^15^ but a study estimating cancer burden in Spain used both discount rate and age weighting in their calculations.^13^ Thus, the characteristics of DALY may differ with the method used and the disease patterns of the studied population.

Cancer incidence data are needed to calculate YLD, but these data are usually available only for a part of a particular country. In the present study, we used data on estimated nationwide incidence for Japan in 2000 from a previous study^10^ by the Japan Cancer Surveillance Research Group, which has provided regular incidence estimates for many years,^10^^,^^17^^,^^18^ using data collected from several population-based cancer registries in Japan.

Public health policies might benefit from using the DALY approach, eg, in estimating potential health improvements gained by appropriate interventions or prevention programs. The DALY can also be used to estimate cancer burden attributable to major risk factors such as tobacco smoking and environmental factors. Evaluation of antismoking programs, for example, might use this summary measure to quantify “effect gain” from potential reductions in cancer incidence and mortality over time, rather than using separate measures.

In summary, we described the national cancer burden in Japan using a measure that reflected both cancer mortality and incidence. We expect the findings to be useful in assessing future changes with respect to mortality and morbidity in Japan.
